# Mediastinal Tracheostoma for Treatment of Tracheostenosis after Tracheostomy in a Patient with Mucopolysaccharidosis-Induced Tracheomalacia

**DOI:** 10.1155/2017/2312415

**Published:** 2017-10-12

**Authors:** Yasuhiro Chikaishi, Kenichi Kobayashi, Shuichi Shinohara, Akihiro Taira, Yusuke Nabe, Shinji Shinohara, Taiji Kuwata, Masaru Takenaka, Soichi Oka, Ayako Hirai, Kazue Yoneda, Koji Kuroda, Naoko Imanishi, Yoshinobu Ichiki, Fumihiro Tanaka

**Affiliations:** Second Department of Surgery, School of Medicine, University of Occupational and Environmental Health, 1-1 Iseigaoka, Yahatanishi-ku, Kitakyushu 807-8555, Japan

## Abstract

**Background:**

Treatment of tracheostenosis after tracheostomy in pediatric patients is often difficult. Mucopolysaccharidosis is a lysosomal storage disease that may induce obstruction of the airways.

**Case Presentation:**

A 16-year-old male patient underwent long-term follow-up after postnatal diagnosis of type II mucopolysaccharidosis. At 11 years of age, tracheostomy was performed for mucopolysaccharidosis-induced laryngeal stenosis. One week prior to presentation, he was admitted to another hospital on an emergency basis for major dyspnea. He was diagnosed with tracheostenosis caused by granulation. The patient was then referred to our institution. The peripheral view of his airway was difficult because of mucopolysaccharidosis-induced tracheomalacia. For airway management, a mediastinal tracheostoma was created with extracorporeal membrane oxygenation. To maintain the blood flow, the skin incision for the mediastinal tracheal hole was sharply cut without an electrotome. The postoperative course was uneventful, and the patient was weaned from the ventilator on postoperative day 19. He was discharged 1.5 months postoperatively. Although he was referred to another institution because of respiratory failure caused by his primary disease 6 months postoperatively, his airway management remained successful for 1.5 years postoperatively.

**Conclusion:**

Mediastinal tracheostomy was useful for treatment of tracheostenosis caused by granulation tissue formation after a tracheostomy.

## 1. Introduction

Treatment of tracheostenosis due to granulation at the tracheostomy site in pediatric patients with congenital disease is often difficult for three main reasons. The first reason is that the tracheal distance is short and the diameter is small in children. The second is that congenital disease is often severe. Finally, patients with congenital disease often develop complications after the treatment of tracheostenosis.

Mucopolysaccharidosis type II (MPS II), also known as Hunter syndrome, is a rare lysosomal storage disorder caused by a deficiency of the lysosomal enzyme iduronate 2-sulfatase. MPS II is associated with a variety of clinical findings including dwarfism, a large head and coarse facial features, stiff and short hands, changes in the spine, a short neck, hepatosplenomegaly, cardiac abnormalities, umbilical hernia, mixed hearing loss, and laryngeal and tracheal abnormalities. Maintaining an airway is extremely important in patients with MPS II and other congenital diseases [1–3].

Patients with MPS II are often tracheostomized [4, 5]. However, many patients experience a difficult clinical course after tracheostomy. Because of the anatomical changes to the trachea in patients with MPS II, the oropharynx and nasopharynx are the main regions that affect respiration. The trachea can become narrowed by accumulation of dermatan sulfate and heparan sulfate, while the oropharynx can be obstructed by tonsillar and adenoid hypertrophy or a relatively large tongue. Moreover, the airway of these patients is often compromised by thickened mucous membranes, copious secretions, recurrent upper respiratory infections, and pneumonia [2, 6, 7].

We herein report a case involving a patient with MPS II who was treated with mediastinal tracheostoma for major airway obstruction by granulation formation at the tracheostomy site.

## 2. Case Presentation

**Figure 1:**
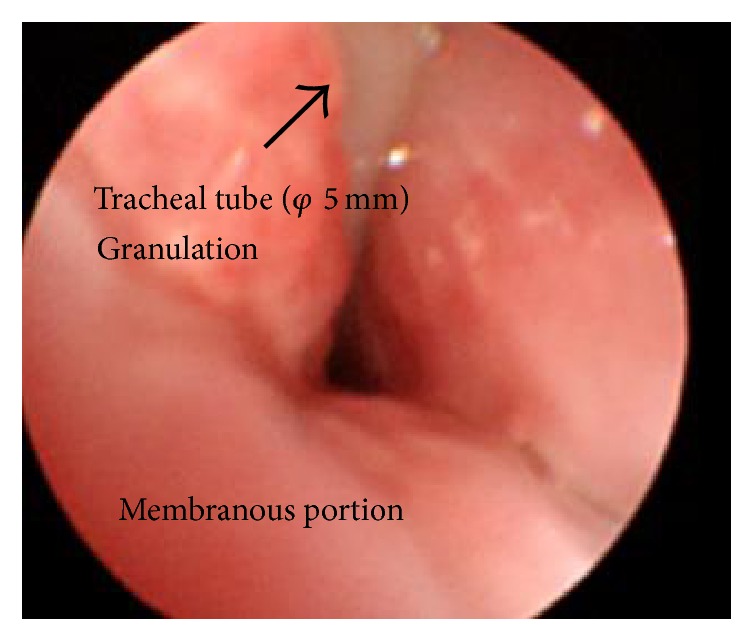
Bronchoscopy showed a stenosed airway. This view was obtained by insertion of the bronchoscope through the nasal cavity.

**Figure 2:**
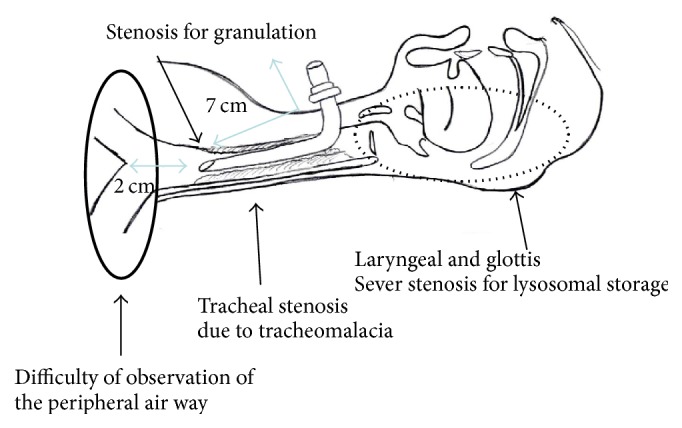
Image of the patient's airway. His larynx was extremely stenotic due to lysosomal storage disease. His trachea was stenosed by granulation tissue secondary to a previous tracheostomy and tracheomalacia. Therefore, peripheral observation was poor.

**Figure 3:**
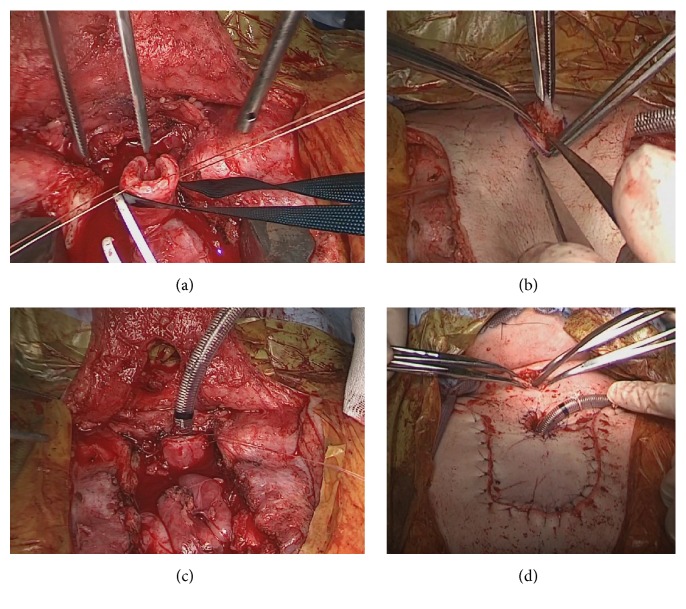
Intraoperative view of the mediastinal tracheostoma. (a) View of the tracheal lumen after tracheal resection. (b) An electric scalpel was not used to create the tracheostoma. (c) View from the back of the U-shaped flap after creation of the tracheostoma. (d) After skin closure following construction of the mediastinal tracheostoma.

**Figure 4:**
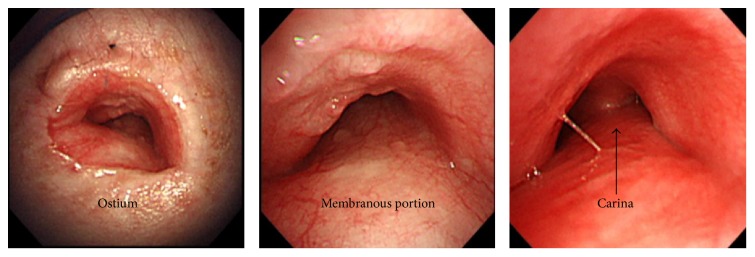
Bronchoscopy findings 6 months after the operation.

A 16-year-old male patient was diagnosed with MPS II in infancy. Five years prior to presentation, when he was 11 years old, tracheostomy was performed to treat an airway obstruction caused by laryngeal stenosis secondary to his primary mucopolysaccharide storage disease. His tracheostomy hole gradually narrowed along with his physical growth, and granulation tissue developed around the tracheostomy hole. One week prior to presentation, at the age of 16 years, he was admitted to another hospital on an emergency basis for major dyspnea. He was diagnosed with tracheostenosis due to granulation at the tracheostomy site and treated with ventilation upon admission. Slight deviation of the position of the tracheal tube obstructed his airway. His previous doctor determined that it was difficult to continue treatment at their institute because no chest surgeon was available. Therefore, the patient presented to our institute by ambulance while undergoing ventilation therapy. We performed a computed tomography scan, which showed obstruction of the airway. The distance from the tip of the tracheal tube to the carina was about 2 cm because the tracheal tube was kept on the distal side of the granulation. Moreover, the patient had pneumonia of the right upper lobe due to the airway obstruction. We performed a bronchoscopic examination through the nasal cavity in the operating room under general anesthesia with the tracheal tube in place because of the risk of suffocation (Figure 1). However, obtaining a peripheral view of the airway was difficult because of macroglossia, swelling of the tonsils, and MPS II-induced tracheomalacia. An image of the patient's airway is shown in Figure 2. We also considered stent placement and laser treatment for airway management but selected the construction of a mediastinal tracheostoma. The mediastinal tracheostoma was created with extracorporeal membrane oxygenation. First, we created a U-shaped flap. The manubrium, clavicle heads, and first and second ventral ribs were resected to facilitate the approach to the anterior mediastinal space. We observed the mediastinum, which exhibited severe adhesion. Second, the innominate vein and artery were sectioned for safe exposure. We exposed the trachea on the head side of the innominate artery and vein. A Metzenbaum scissors was used to cut the trachea at the minimum required length for construction of the mediastinal tracheostoma. After we confirmed that no granuloma was present in the tracheal lumen on the peripheral side (Figure 3(a)), we intubated the trachea on the surgical field side. Third, we marked the mediastinal tracheal hole on the U-shaped flap. To maintain blood flow, the skin incision for the mediastinal tracheal hole was cut using a sharp scalpel and Metzenbaum scissors, without an electrotome (Figures 3(b) and 3(c)). The skin and trachea were closed with 4-0 polypropylene interrupted sutures. Mediastinal tracheostomy was performed with an inlay thymus graft between the innominate vein and trachea (Figure 3(d)). The postoperative course was uneventful, and the patient was weaned from the ventilator on postoperative day 19. He was discharged at 1.5 months postoperatively. Although he was referred to another institution for respiratory failure associated with his primary disease 6 months postoperatively, his airway management was successful for 1.5 years postoperatively (Figure 4).

## 3. Discussion

In general, treatment of tracheostenosis due to granulation formation after tracheostomy in patients with congenital disease is difficult. The most notable aspect of the present report is the airway management technique used for a complicated case of tracheomalacia. When examining patients with tracheostenosis after tracheostomy, we select either surgery (such as tracheoplasty [8]) or conservative therapy (such as stent placement or laser treatment [9]) according to the shape, location, and extent of the stenosis. Among these treatments, tracheoplasty, stent placement, or laser therapy is usually selected. However, it is rare case that a mediastinal tracheostoma is constructed for treatment of tracheostenosis after tracheostomy, as in the present case.

In our patient, we did not perform a general intervention for airway management, such as tracheoplasty, stent placement, or laser therapy, for the following reasons. A peripheral view of his trachea was difficult because of the extent of stenosis, MPS II-induced tracheomalacia, and small diameter of the airway. Moreover, it was difficult to insert a bronchoscope because of the stenosis at the entrance of the tracheostoma and narrowing of the larynx. A surgical procedure such as tracheoplasty would have required a peripheral approach to avoid restenosis or anastomotic failure. It would have been difficult to select the most appropriate type of stent because of the unconfirmed length and diameter of the granulation. Moreover, we were unable to insert an appropriate bronchoscope. Laser therapy is difficult because of the risk of surgical fire [10] and narrow airway diameter caused by granulation and tracheomalacia. For these reasons, we selected construction of a mediastinal tracheostoma including laryngotracheal separation, which is a rare airway management technique.

Through the establishment of a mediastinal tracheostoma, we were able to maintain the tracheal airway with no stenosis in the periphery, separate from the area of granulation. Moreover, it was possible to more safely manage the tracheal tube because the diameter of the tracheostoma was secured. However, there was still a risk of stenosis of the peripheral airway associated with the resected part of the trachea. PCPS was needed to prevent complications during intraoperative intubation. We currently use PCPS to manage high-risk airway stenosis as in the present case. From a technical viewpoint, during the skin incision for the tracheal hole, we do not use material that may cause burns on the skin because the blood flow must be maintained. Instead, we sharply cut the skin that is anastomosed to the trachea using a sharp scalpel or scissors only, as described in the present case.

In pediatric patients with congenital diseases such as MDS II, tracheostenosis after tracheostomy is a complication that must be carefully managed. Airway management using construction of a mediastinal tracheostoma can be a sufficient treatment option.
